# Structural and Functional Abnormalities in Knee Osteoarthritis Pain Revealed With Multimodal Magnetic Resonance Imaging

**DOI:** 10.3389/fnhum.2021.783355

**Published:** 2021-11-29

**Authors:** Hua Guo, Yuqing Wang, Lihua Qiu, Xiaoqi Huang, Chengqi He, Junran Zhang, Qiyong Gong

**Affiliations:** ^1^Department of Rehabilitative Medicine, West China Hospital, Sichuan University, Chengdu, China; ^2^Tsinghua University, Beijing, China; ^3^Radiology Department, The Second People’s Hospital of Yibin, Yibin, China; ^4^Huaxi MR Research Center (HMRRC), Department of Radiology, West China Hospital of Sichuan University, Chengdu, China; ^5^School of Electrical Engineering, Sichuan University, Chengdu, China

**Keywords:** knee osteoarthritis pain, structural MRI, resting-state fMRI, GMV, ALFF

## Abstract

The knee osteoarthritis (KOA) pain is the most common form of arthritis pain affecting millions of people worldwide. Long-term KOA pain causes motor impairment and affects affective and cognitive functions. However, little is known about the structural and functional abnormalities induced by long-term KOA pain. In this work, high-resolution structural magnetic resonance imaging (sMRI) and resting-state functional MRI (rs-fMRI) data were acquired in patients with KOA and age-, sex-matched healthy controls (HC). Gray matter volume (GMV) and fractional amplitude of low-frequency fluctuation (fALFF) were used to study the structural and functional abnormalities in patients with KOA. Compared with HC, patients with KOA showed reduced GMV in bilateral insula and bilateral hippocampus, and reduced fALFF in left cerebellum, precentral gyrus, and the right superior occipital gyrus. Patients with KOA also showed increased fALFF in left insula and bilateral hippocampus. In addition, the abnormal GMV in left insula and fALFF in left fusiform were closely correlated with the pain severity or disease duration. These results indicated that long KOA pain leads to brain structural and functional impairments in motor, visual, cognitive, and affective functions that related to brain areas. Our findings may facilitate to understand the neural basis of KOA pain and the future therapy to relieve disease symptoms.

## Introduction

Osteoarthritis is a degenerative joint disease with high incidence and mainly occurs in the elderly population. The knee osteoarthritis (KOA) is one of the most common osteoarthritis which affects millions of people worldwide. Knee pain is a main symptom of KOA which results in decreasing mobility to make the quality of life of the patients worse (Kloppenburg and Berenbaum, 2020). KOA pain is a ubiquitous and chronic pain which leads to restricted movement, sleep disturbance, and psychosocial disability (Ferket et al., 2017; Zhaoyang et al., 2020). Typically, KOA pain is worsened with activities, such as walking or climbing steps, and relieved with rest (Losina et al., 2016). Kaplan et al. (2019) disclosed that the degree of pain does not always predict the extent of joint damage or the presence of active inflammation, which suggests the spread of central sensitizations as the underlying primary mechanisms involved in KOA pain.

The pathogenic mechanisms of pain in KOA may be related to central sensitization mechanisms (Kaplan et al., 2019). KOA can lead to persistent chronic pain affecting nervous system structure and function (Gollub et al., 2018; Zhang et al., 2020). With the development of magnetic resonance imaging (MRI), it is able to non-invasively investigate the brain structure and function and to map the brain connectivity and network *in vivo* (Xu et al., 2019, 2020; Becq et al., 2020; Li et al., 2021; Wang et al., 2021). The high-resolution sMRI is mainly used to characterize the brain morphological properties (Wang et al., 2019). The rs-fMRI allows us to study the intrinsic functional activity pattern of brain. Derived from rs-fMRI, many measures including functional connectivity (Biswal et al., 1995), regional homogeneity (Zang et al., 2004), functional connectivity pattern homogeneity (Wang et al., 2018), functional connectivity density (Tomasi and Volkow, 2010), and amplitude of low-frequency fluctuation (ALFF) (Zou et al., 2008) were proposed and applied to characterize the brain functional couplings, integration, or activity (Zou et al., 2016; Sun et al., 2018; Wang et al., 2018). In all of these measures, ALFF is a widely used index to reflect the functional activity level. The ALFF has been widely used to study the functional activities in both healthy and diseased brain (Fu et al., 2018; Wang et al., 2020). However, whether/how long-term KOA induces brain structural and functional changes is still an open problem.

To reveal the structural and functional abnormalities in patients with KOA, structural and rs-fMRI data were acquired from 13 individuals with KOA and 13 age-, sex-, and education-matched healthy controls (HC). Voxel-based morphological analysis of GMV and voxel-wise analysis of ALFF were performed. We hypnotized that patients with KOA may show structural or functional abnormalities in sensorimotor, emotion, and cognition related brain areas.

## Materials and Methods

### Participants

Thirteen right-handed women patients with chronic KOA pain (mean age = 55.5 years, standard deviation = 5.5 years) were recruited from the West China Hospital, Sichuan University, Chengdu, China. Thirteen age- and sex-matched HC were also included (mean age = 53.9 years, standard deviation = 5.6 years) (Table 1). The inclusion criteria for KOA were as follows: (1) without neurological or psychiatric disorders; (2) without a history of medication (within 1 month) or alcohol abuse (within 1 year); (3) with typical KOA symptoms as diagnosed by X-ray; (4) without contraindications for MRI; and (5) without conflicting medications. The inclusion criteria for HC were as follows: (1) no chronic medications and no history of chronic pain; (2) no neurological or psychiatric disorders; (3) no alcohol abuse within one year; and (4) no contraindications to MRI. The written informed consent of each subject was obtained, and this work was approved by the local ethical committee of West China Hospital of Sichuan University and in accordance with the Declaration of Helsinki.

**TABLE 1:** Characteristics of demographic and clinical variables.

	KOA (*n* = 13)	HC (*n* = 13)	*p*-value
Age (years)	55.5 ± 5.5	53.9 ± 5.6	0.95
Gender (male/female)	0/13	0/13	0.97
Education (years)	9.97 ± 4.18	9.66 ± 3.65	0.68
Duration (years)	4.73 ± 4.23	0	<0.01
VAS: left knee	26 ± 19.85	0	<0.01
right knee	63.08 ± 9.25	0	<0.01
HSS: left knee	91.23 ± 7.42	100	<0.01
right knee	73.62 ± 4.11	100	<0.01

*Values are mean ± standard deviation; VAS, visual analog scale; HSS, Hospital for Special Surgery scale (HSS); KOA, knee osteoarthritis; HC, healthy controls.*

### Hospital for Special Surgery Scale and Visual Analog Scale Assessment

The clinical symptoms were assessed using visual analog scale (VAS) and Hospital for Special Surgery scale (HSS) (Tian et al., 2020) by two experienced physicians when the subjects were loading (standing). The loading was defined as the self-standup balance between the two legs, that is, the subjects are standing on their own. According to the standard of HSS knee function score (very good: ≥85, good: 70–84, in general: 60–69, poor: ≤59), all patients were divided into four levels. The difference in HSS between the right and left knee (D_*HSS*_) was compared. The VAS was used to assess the pain severity of knees for all the subjects (scale ranges from 0 to 100; 0 = no pain; 100 = worst imaginable pain) (Király et al., 2020). Subjects were evaluated when they had stable pain, and the difference in the VAS scores between the right and left knee was also compared.

### Magnetic Resonance Imaging Acquisition

Magnetic resonance imaging data was scanned on a 3T whole-body MRI scanner (Siemens Trio system, Erlangen, German) at the MRI Research Center of West China Hospital of Sichuan University. Before scanning, all the subjects were asked to keep their eyes closed with clear thoughts and not to fall asleep. The rs-fMRI data were acquired with a T2* weighted single-shot echo-planar imaging (EPI) sequence using an eight-channel head coil. The acquisition parameters were as follows: repetition time (TR) = 2000 ms, echo time (TE) = 30 ms, flip angle = 90°, field of view (FOV) = 24 cm^2^ × 24 cm^2^, matrix = 64 × 64, 30 transverse slices covering the whole head, slice thickness = 5.0 mm, voxel size = 3.75 mm^3^ × 3.75 mm^3^ × 5 mm^3^, and 180 volumes. A high-resolution 3D T1-weighted image was also acquired using a spoiled gradient recalled (SPGR) sequence with the following parameters: TR = 8.5 ms, TE = 3.4 ms, flip angle = 12°, FOV = 24 × 24 cm^2^, scan matrix = 256 × 256, 156 slices, voxel dimensions = 0.94 × 0.94 × 1 mm^3^.

### Voxel-Based Morphometry Analysis

The high-resolution 3D anatomical images of all subjects were processed using VBM8 and SPM8 toolkits.^1^ The sMRI image was first segmented into gray matter, white matter, and cerebrospinal fluid. Then, all the segmented images were registered into MNI space using DARTEL method (Ota et al., 2015; Igata et al., 2017; Wang et al., 2017, 2018; Wu et al., 2017; Gao et al., 2020). Modulated GMV for each subject was obtained and was smoothed using Gaussian kernel with FWHM = 10 mm. To identify structural abnormality in patients with KOA, two-sample *t*-test was applied to test the differences in GMV and corrected with modified Alphasim correction method with *p* < 0.05 (voxel *p* < 0.001).

### Resting-State Functional Magnetic Resonance Imaging Pre-processing

Resting-state fMRI data pre-processing was carried out using DPARSF software. The first five volumes were discarded to ensure magnetization equilibrium. The remaining 175 volumes were realigned to the first volume to correct the effect of head motion. Then, the fMRI images were warped into the MNI space with a resolution of 3 × 3 × 3 mm^3^ with T1 images and were spatially smoothed by Gaussian kernel with FWHM = 4 mm. The smoothed volumes were detrended and finally were used to calculate the fALFF.

### Functional Activity Analysis

The brain functional activity was characterized using fALFF in this work. The fALFF score was the ratio between the power of low-frequency components (0.01–0.08 Hz) and the power of all-frequency components, which described the contribution of the low-frequency components to the all-frequency bands. For statistical analyses, fALFF was normalized by divided the whole brain of ALFF in each subject. The whole brain voxel-wise statistical analysis with two-sample *t*-test was performed to identify the functional activity difference in fALFF between patients with KOA and HC, and the statistical result was corrected using modified Alphasim correction method with *p* < 0.05 (voxel *p* < 0.001).

### Correlation Analysis

After obtaining the regions with abnormal GMV or fALFF, the regional average of GMV or fALFF was calculated for each subject and used to explore the relationship with clinical symptoms. To investigate the relationship between structural and functional abnormalities with pain intensity, joint function, and disease duration, correlation analyses were performed using Pearson’s correlation. The significant level was set at *p* < 0.05 uncorrected.

**FIGURE 1:**
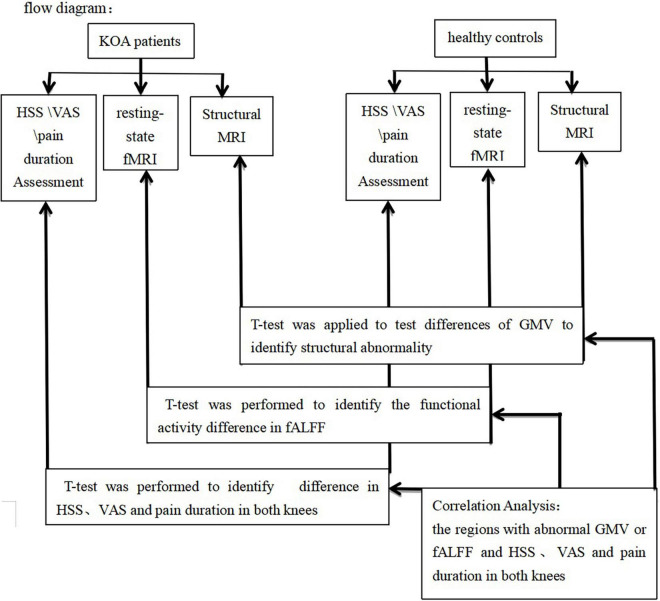
Flow diagram.

Flow diagram was shown in Figure 1.

## Results

As shown in Table 1, no significant differences in age and sex were found between patients with KOA and HC. In patients with KOA, the VAS scores were significantly different between their left knees and right knees (*p* < 0.01). The level of HSS was also significantly different between their left knees and right knees (*p* < 0.01).

### Changed Gray Matter Volume

**FIGURE 2:**
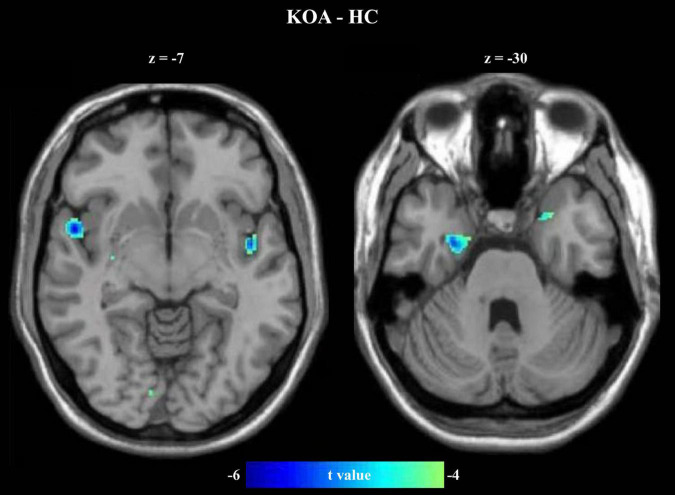
Reduced GMVs in patients with KOA. Compared with HC, the patients with KOA pain showed lower GMV in the bilateral insula and hippocampus.

Compared with the HC, the patients with KOA exhibited reduced GMV in the bilateral insula and bilateral hippocampus (Figure 2 and Table 2).

**TABLE 2:** Reduce volume of gray matter in patients with KOA pain.

Regions	MNI (Peak)	*t* value (Peak)	*p-* value (Peak)
Left insula	−45, −5, −6	−4.63	5.3e-5
Right insula	53, 6, −8	−5.03	1.9e-5
Left hippocampus	−21, 8, −30	−4.44	8.6e-5
Right hippocampus	29, -6, −30	-5.19	1.3e-5

### Abnormal Fractional Amplitude of Low-Frequency Fluctuation in Knee Osteoarthritis

**FIGURE 3:**
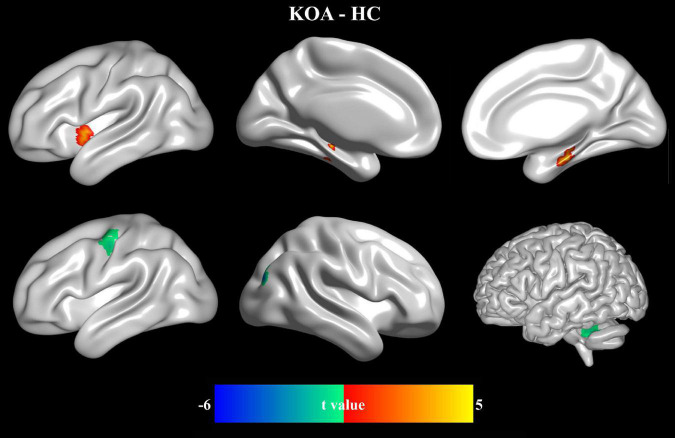
Abnormal resting-state functional activities in patients with KOA. Comparing with HC, the patients with KOA showed higher fALFF in left insula and bilateral hippocampus while lower fALFF in left precentral gyrus, left cerebellum, and right superior occipital gyrus.

As shown in Figure 3 and Table 3, patients with KOA exhibited higher fALFF values in the left insula and bilateral hippocampus while lower fALFF values in the left cerebellum, left precentral gyrus, and right superior occipital gyrus compared with the control group (Figure 3 and Table 3).

**TABLE 3:** Comparison of fALFF measurements in patients with KOA and control subjects.

Regions	Coordinate	*t* value	*p-*value
Left insula	−39, 6, −3	3.17	0.002
Hippocampus	15, −12, −24	4.11	0.0002
Left cerebellum	−42, −45, −24	–4.17	0.0002
Left precentral gyrus	−39, −9, 45	–5.5	0.000005
Right superior occipital gyrus	33, −84, 33	–4.45	0.00008

### Clinical Correlation Analyses

**FIGURE 4:**
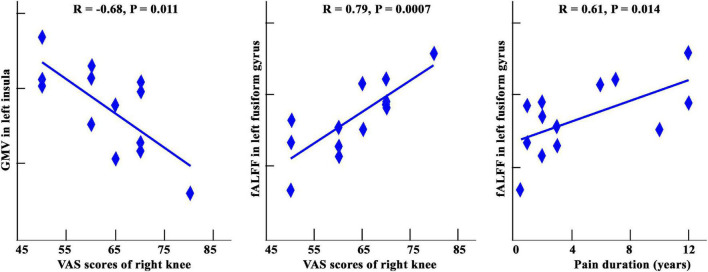
A: Correlation analysis among GMV, fALFF, and clinical assessments in patients with KOA. Significantly negative correlation between GMV of left insula and VAS scores of right knees was found. The significantly positive correlations between fALFF values in left fusiform gyrus and VAS score and pain duration were also found.

As shown in Figure 4, GMVs in the left insula showed a significantly negative correlation with the VAS for the right knee (*r* = −0.68, *p* = 0.011). A significant positive correlation was found between VAS scores in the right knees and fALFF in left fusiform gyrus (*r* = 0.79, *p* = 0.0007). A significant positive correlation was also found between pain duration and fALFF in the left fusiform gyrus (*r* = 0.61, *p* = 0.014). No other significant correlations were found.

## Discussion

This work had three significant findings: (1) patients with chronic KOA pain exhibited reduced GMVs in the bilateral insula and hippocampus; (2) compared with the control group, the patient with KOA showed increased fALFF values in the left insula and bilateral hippocampus whereas decreased fALFF values in the left cerebellum, left precentral gyrus, and right superior occipital gyrus; and (3) the GMV in left insula and the fALFF value in left fusiform gyrus showed significant correlation with pain intensity, and the fALFF of left fusiform gyrus also showed significant correlation with disease duration in patients with KOA. These findings indicate that the left insula may be important for pain processing. More importantly, our findings highlight the key role of left fusiform gyrus in neuropathology of KOA.

The work of spontaneous fluctuations in the brain of patients with KOA reported that the involved networks in the central nervous system are associated with negative emotions and memories of pain perceptions and movement. KOA can lead to persistent chronic pain affecting nervous system function and resulting in secondary changes in brain activity which can be detected by electrophysiological or imaging techniques (Gollub et al., 2018).

Moreover, the orbitofrontal cortex in pain patients have exhibited abnormal activity during a cognitive task, which suggests that pain disturbs the normal functioning of orbitofrontal cortex during emotional modulation (Petrovic et al., 2000). In this work, the orbitofrontal cortex was activated during the anticipation of pain stimulus, which has been reported as representative pain-related expectation regions (Ushio et al., 2020). Furthermore, cognitive control can reduce pain and in part has been attributed to a brain network which comprises prefrontal regions including orbitofrontal cortex, the anterior insula, anterior cingulate cortex (ACC), and brainstem regions (Seminowicz and Moayedi, 2017).

### Hippocampus and Insula in Knee Osteoarthritis Pain

The hippocampus is one of the major areas that links affective states, memory processing, pain processing, memory of pain stimulation, and the development of fear-initiated avoidance (Price and Inyang, 2015; Schmidt et al., 2019). Abnormal hippocampal structure and function that occur in chronic pain has been widely reported. Osteoarthritis was reported to be associated with a faster decline in hippocampal volumes in cognitively normal older people (Li et al., 2020). Abnormal hippocampal function in chronic pain might reorganize with neuropathic pain (Jacobs et al., 2020). Strong activation during a pricking pain condition was found in the hippocampal formation, which is related to the aspects of nociceptive processing (Veldhuijzen et al., 2009). The enhanced spontaneous fluctuation of hippocampus in patient with KOA might originate from long-term memory formation and deepening of chronic KOA pain (Wang et al., 2011). Fasick et al. (2015) found that an inflammatory response in the hippocampus might contribute to the enhancement of pain sensitivity. Using positron emission tomography and computed tomography (PET-CT), Yang et al. (2012) found that migraine patients exhibited reduced metabolism in the hippocampus following traditional acupuncture therapy suggested that pain-related emotions and memories faded and disappeared as migraine remission occurs. These results collectedly demonstrated that abnormal GMV and fALFF in hippocampus found in our work may be associated with pain-related emotions and memory deficits (Ezzati et al., 2014).

The patients with chronic KOA pain also exhibited reduced GMVs in the insula. Our findings were consistent with a recent work which showed reduced GMV in insular cortex in patients with persistent subacute back pain (Yang and Chang, 2019). Altered connectivity of the right anterior insula driving the pain connectome changes in chronic knee osteoarthritis was also observed (Cottam et al., 2016). The posterior insula was a part of the lateral pain system, which was critical for the perception, encoding, modulation, and chronification of pain and also the formation of pain experiences and the emotional aspects of pain (Lu et al., 2016; Cottam et al., 2018). Thus, the reductions in the GMV of the insula might be associated with sustained abnormal activity in the insula that was evoked by continuous KOA pain. In addition, the GMV in insula was negatively correlated with KOA pain severity. Given that insula anatomically connected with thalamus and supplementary motor area (Mertens et al., 2015), the correlation results further confirmed that insula may be associated with abnormal movement or motor preparation triggered by limb pain (Brown et al., 2014; Wang et al., 2015).

### Fusiform Gyrus in Knee Osteoarthritis Pain

Fusiform gyrus located in the ventral of temporal cortex was found to show that activation increases in specific phobias probably due to increased processing of the cue and expectation of behaviorally relevant sensory input (Landgrebe et al., 2008). Ruscheweyh et al. (2018) studied the relationship between GMV and pressure pain thresholds, self-rated pain sensitivity, and found that pain sensitivity questionnaire scores were positively correlated with GMV in fusiform gyrus. Ma et al. (2018) found that migraineurs with comorbid depression had different developmental trajectories in the right fusiform, which were associated with recognizing, transmitting, controlling, and remembering pain and emotion. In this work, we found that the fALFF values in the left fusiform of patients with KOA were significantly positively related to both KOA pain duration and pain intensity in right knees, which indicates that the fusiform gyrus might be a characteristic brain area allowing for the diagnostic imaging of neural mechanism for KOA pain. In conclusion, based on the results of correlation analyses and spontaneous activity, we proposed that the fusiform gyrus should be regarded as a critical area for assessing the mechanism of central nervous system sensitivity to KOA chronic pain.

### Other Brain Areas in Knee Osteoarthritis Pain

Abnormal functional activities in left cerebellum, left precentral gyrus, and right superior occipital gyrus were found. The cerebellum is involved in autonomic control, cognition, and affect, as well as in sensorimotor control (Adamaszek et al., 2017). It appears to play a crossmodal modulatory role in relation to pain with noxious stimuli impacting the processing of generalized aversions and sensorimotor adaptations to pain (Mehnert et al., 2017). Cerebellar activity changes during noxious stimuli have been considered to represent offensive motor responses. Left precentral gyrus is important not only in executive functions but also in pain process of antinociceptive effects due to its connections with other subcortical areas for the modulation of pain and inducing pain chronification (Ong et al., 2019). The decreased fALFF value in left precentral gyrus may imply that the long-term disability in the right knee would alter the intrinsic patterns of contralateral movement control among the patients with KOA. In addition, the superior occipital lobe that showed decreased fALFF in patients with KOA plays a critical role in the allocation of visuospatial attention (Sengupta et al., 2018). The decreased fALFF value in this area indicates the impaired reallocation of visuospatial attention for self-regulation in patients with KOA.

### Limitations

The sample size was small due to the exclusion of the patients with KOA who had received standard therapy and patients who had reported higher pain intensity in the left than in the right knee. Moreover, our work was conducted without the treatment for a month making it more challenging to find suitable subjects. Larger sample sizes are required to generalize our findings in this work.

## Conclusion

Regional structural and functional abnormalities have been found in women with KOA using structural and rs-fMRI compared with that in healthy volunteers. The GMV analysis revealed abnormal pain perception and memory in patients with KOA. The spontaneous brain activity analyses revealed that patients with KOA exhibited two different patterns, a pain-related pattern with higher fALFF and a movement control-related pattern with lower fALFF. The function alternation of fusiform gyrus can be used as a potential biomarker for KOA severity.

## Data Availability Statement

The raw data supporting the conclusions of this article will be made available by the authors, without undue reservation.

## Ethics Statement

The studies involving human participants were reviewed and approved by West China Hospital of Sichuan University. The patients/participants provided their written informed consent to participate in this study.

## Author Contributions

All authors listed have made a substantial, direct, and intellectual contribution to the work, and approved it for publication.

## Conflict of Interest

The authors declare that the research was conducted in the absence of any commercial or financial relationships that could be construed as a potential conflict of interest.

## Publisher’s Note

All claims expressed in this article are solely those of the authors and do not necessarily represent those of their affiliated organizations, or those of the publisher, the editors and the reviewers. Any product that may be evaluated in this article, or claim that may be made by its manufacturer, is not guaranteed or endorsed by the publisher.

## Footnotes

1
http://www.fil.ion.ucl.ac.uk/spm
